# What Is Driving the Need for Home-Delivered Meals? Findings From the Deliver-EE Study

**DOI:** 10.1093/geroni/igaf122.475

**Published:** 2025-12-31

**Authors:** Kali Thomas, Bernadette Wright, Julie Locher

## Abstract

Home-delivered meals are provided to 2.2 million older adults each year. Prior research has documented the benefit of meals for older adults’ health and nutrition; however, the funding for home-delivered meals has not kept pace with the need. This symposium leverages findings from the Deliver-EE study to describe the varied reasons that older adults seek out nutrition support and their experiences after receiving home-delivered meals. Deliver-EE is a pragmatic clinical trial funded by the Patient-Centered Outcomes Research Institute and operates in partnership with Meals on Wheels America and the AARP Foundation. Deliver-EE is enrolling 2,300 older adults ages 66 years and older from waitlists at 14 Meals on Wheels programs located in California, Texas, Maryland, North Carolina, South Carolina, Tennessee, Illinois and Florida. This panel features a series of presentations focused on the pre-intervention experiences of participants, bridging their insights from quantitative administrative and baseline survey data (n = 1775) with qualitative interviews (n = 99). The first presenter highlights challenges of diet quality and food insecurity among home-delivered meal recipients; The second discusses financial strain and related coping strategies observed in this sample; The third shares findings about this sample’s social isolation and loneliness; and The fourth describes the benefits and unmet needs that both caregivers and older adults report from receiving home-delivered meals. Our discussant situates the findings in the landscape of nutrition research and shares reactions to findings in the context of the current and future need for home-delivered meals.

